# Noninvasive k_3_ estimation method for slow dissociation PET ligands: application to [^11^C]Pittsburgh compound B

**DOI:** 10.1186/2191-219X-3-76

**Published:** 2013-11-16

**Authors:** Koichi Sato, Kiyoshi Fukushi, Hitoshi Shinotoh, Hitoshi Shimada, Shigeki Hirano, Noriko Tanaka, Tetsuya Suhara, Toshiaki Irie, Hiroshi Ito

**Affiliations:** 1 Molecular Imaging Center, National Institute of Radiological Sciences, 4-9-1 Anagawa, Inage-ku, Chiba 260-8555, Japan; 2 Department of Psychiatry, Teikyo University Chiba Medical Center, 3426-3 Anesaki, Ichihara-shi, Chiba 299-0111, Japan; 3 Neurology Chiba Clinic, 1-2-12 Benten, Chuo-ku, Chiba 260-0045, Japan; 4 Section for Human Neurophysiology, Research Center for Frontier Medical Engineering, Chiba University, 1-33 Yayoi-cho, Inage-ku, Chiba 263-8522, Japan; 5 Department of Neurology, Graduate School of Medicine, Chiba University, 1-8-1 Inohana, Chuo-ku, Chiba 260-8677, Japan; 6 Bureau of Social Welfare and Public Health, Tokyo Metropolitan Government, 2-8-1 Nishi-shinjuku, Shinjuku-ku, Tokyo 163-8001, Japan

**Keywords:** [^11^C]Pittsburgh compound B, Alzheimer's disease, Kinetic modeling, PET quantification, Reference tissue, Slow dissociation ligand

## Abstract

**Background:**

Recently, we reported an information density theory and an analysis of three-parameter plus shorter scan than conventional method (3P+) for the amyloid-binding ligand [^11^C]Pittsburgh compound B (PIB) as an example of a non-highly reversible positron emission tomography (PET) ligand. This article describes an extension of 3P + analysis to noninvasive ‘3P++’ analysis (3P + plus use of a reference tissue for input function).

**Methods:**

In 3P++ analysis for [^11^C]PIB, the cerebellum was used as a reference tissue (negligible specific binding). Fifteen healthy subjects (NC) and fifteen Alzheimer's disease (AD) patients participated. The *k*_3_ (index of receptor density) values were estimated with 40-min PET data and three-parameter reference tissue model and were compared with that in 40-min 3P + analysis as well as standard 90-min four-parameter (4P) analysis with arterial input function. Simulation studies were performed to explain *k*_3_ biases observed in 3P++ analysis.

**Results:**

Good model fits of 40-min PET data were observed in both reference and target regions-of-interest (ROIs). High linear intra-subject (inter-15 ROI) correlations of *k*_3_ between 3P++ (*Y*-axis) and 3P + (*X*-axis) analyses were shown in one NC (*r*^2^ = 0.972 and slope = 0.845) and in one AD (*r*^2^ = 0.982, slope = 0.655), whereas inter-subject *k*_3_ correlations in a target region (left lateral temporal cortex) from 30 subjects (15 NC + 15 AD) were somewhat lower (*r*^2^ = 0.739 and slope = 0.461). Similar results were shown between 3P++ and 4P analyses: *r*^2^ = 0.953 for intra-subject *k*_3_ in NC, *r*^2^ = 0.907 for that in AD and *r*^2^ = 0.711 for inter-30 subject *k*_3_. Simulation studies showed that such lower inter-subject *k*_3_ correlations and significant negative *k*_3_ biases were not due to unstableness of 3P++ analysis but rather to inter-subject variation of both *k*_2_ (index of brain-to-blood transport) and *k*_3_ (not completely negligible) in the reference region.

**Conclusions:**

In [^11^C]PIB, the applicability of 3P++ analysis may be restricted to intra-subject comparison such as follow-up studies. The 3P++ method itself is thought to be robust and may be more applicable to other non-highly reversible PET ligands with ideal reference tissue.

## Background

Various reversible-type radioligands have been developed for *in vivo* neuroreceptor study with positron emission tomography (PET). Both arterial blood sampling and long dynamic PET scan, up to 120 min, are required for standard nonlinear least-squares (NLS) analysis to estimate *K*_1_ to *k*_4_ in the two-tissue compartment four-parameter model (4P model): *K*_1_ represents the blood-to-brain transport constant, *k*_2_ represents the brain-to-blood transport constant, *k*_3_ represents the first-order association rate constant for specific binding, and *k*_4_ represents the dissociation rate constant for specific binding. The *k*_3_ represents *B*_max_·*k*_on_, where *B*_max_ is maximum receptor density and *k*_on_ is the *in vivo* association rate constant. Since *k*_3_ represents available receptors for the PET ligand, it is the target parameter of major interest in most PET studies. However, quantification of *k*_3_ in the 4P model is often difficult because of uncertainty of the *k*_4_ estimate and high correlation between the *k*_3_ and *k*_4_ estimates. As surrogate parameters for *B*_max_, binding potential and distribution volume have been widely used [1-4]. Several reference tissue methods have also been developed [5-10].

Irreversible (enzyme-substrate type) radiotracers [^11^C]methylpiperidin-4-yl acetate and propionate have been developed for the measurement of cerebral acetylcholine esterase activity using PET [11,12]. In this case the two-tissue compartment three-parameter (*K*_1_ to *k*_3_) model (3P model) was used to estimate *k*_3_, which is an index of acetylcholine esterase activity. In the 3P model, the precision of *k*_3_ estimate is usually higher than in the 4P model, in spite of shorter PET scan time (40 to 60 min), since there is no need of *k*_4_ estimation in the 3P model.

We have previously defined two mathematical functions, the information density function and information function, which are useful for model selection and optimization of scan time in PET [13]. Based on simulations using both functions, we proposed a new method (3P + method) for quantification of *k*_3_ for moderately reversible ligands. ‘3P+’ means three-parameter model plus short PET scan. In this method, the 3P model (*k*_4_ = 0 model) was applied to the early-phase PET data (up to 30 to 40 min) from reversible ligands with moderate *k*_4_ (moderately reversible ligands). Although the 3P + method was not always developed for a specific ligand, the amyloid-binding radiotracer [^11^C]Pittsburgh compound B (PIB) was used as an example for the moderately reversible ligands (*k*_4_ = 0.018/min). The 3P + method afforded a more stable *k*_3_ estimate than the standard 90-min 4P analysis. However, there is still the drawback of the necessity for arterial blood sampling and radiometabolite analysis, which may restrict the widespread use of this method in daily clinical practice.

In this article, we propose a noninvasive 3P++ analysis using [^11^C]PIB. 3P++ means 3P + analysis plus use of a reference tissue for input function. To validate the proposed method, the linear correlations of *k*_3_ estimates were evaluated between 40-min 3P++ and 3P + analyses, as well as between 3P++ and 90-min 4P analyses in clinical PET studies. In addition, simulation studies were performed to explain *k*_3_ biases observed in the 3P++ analysis.

## Methods

### Theory

#### Assumptions in 3P++ analysis

The following are assumptions used in 3P++ analysis:

•Assumption 1 (on the nature of radioligand used): We apply 3P++ analysis only to moderately reversible or nearly irreversible radioligands (*k*_4_ ≤ 0.03/min), but exclude highly reversible ligands. [^11^C]PIB is an example of moderately reversible ligands (*k*_4_ = 0.018/min).

•Assumption 2 (on the duration time of PET scan): We use early-phase PET data in the curve fitting. In [^11^C]PIB, dynamic PET data during 0 to 40 min was described well with the 3P model, since the effect of the *k*_4_ process on PET data was negligible within these early-phase kinetics [13].

•Assumption 3 (on the specific binding in the reference tissue, *k*_3r_): Specific binding of radioligand is negligible in the reference tissue (*k*_3r_ = 0). In [^11^C]PIB, the gray matter of the cerebellum is usually used as a reference tissue for input function [14]. We apply the one-tissue compartment two-parameter (*K*_1_, *k*_2_) model (2P model) to the reference tissue.

#### Working equation for 3P++ analysis

The working equation for the 3P++ analysis has been reported [15]:

(1) $$\begin{array}{ll} C_{t}\left(t\right) & =R_{1}\left[\delta \left(t\right)+\frac{k_{2r}k_{3}}{k_{2}+k_{3}}+\left(k_{2r}-k_{2}-\frac{k_{2r}k_{3}}{k_{2}+k_{3}}\right)e^{-\left(k_{2}+k_{3}\right)t}\right]⊗C_{r}(t)\\ & =R_{1}C_{r}\left(t\right)+\frac{R_{1}k_{2r}k_{3}}{k_{2}+k_{3}}\int_{0}^{t}C_{r}\left(\tau \right)\mathrm{d\tau}-\frac{R_{1}k_{2}\left(k_{2}+k_{3}-k_{2r}\right)}{k_{2}+k_{3}}\\ & \;x\int_{0}^{t}e^{-\left(k_{2}+k_{3}\right)\left(t-\tau \right)}C_{r}\left(\tau \right)\mathrm{d\tau}, \end{array}$$

where *C*_*t*_(*t*) is the radioactivity concentration in the target tissue and *C*_*r*_(*t*) is that in the reference tissue; *k*_2r_ is the *k*_2_ in the reference tissue and ⊗ is the convolution integral. The rate of tracer penetration into the target tissue is obtained as the relative value *R*_1_, which is the ratio of target *K*_1_ to reference *K*_1_.

### Clinical PET study

#### Human subjects

Two groups of subjects, a normal control (NC) group and an Alzheimer's disease (AD) group, participated in the current study with written informed consent. The NC group consisted of 15 healthy subjects (age ranging from 48 to 90 years, 66.7 ± 11.5 years (mean ± SD); eight males and seven females) without a history of central nervous system diseases or psychiatric disorders, and the AD group consisted of 15 patients (ages 55 to 85, 68.9 ± 9.6 years; four males and 11 females) diagnosed as probable AD according to the criteria of the National Institute of Neurological and Communication Disorders, Alzheimer's Disease and Related Disorders Association [16]. The study was approved by the Institutional Review Board of the National Institute of Radiological Sciences.

#### Radiochemical synthesis

[^11^C]PIB was synthesized by the reaction of 2-(4′-aminophenyl)-6-hydroxy-benzothiazole and [^11^C]methyl triflate [17]. The product had radiochemical purity greater than 95.4%. Specific activity was in the range of 56.3 to 285.3 GBq/μmol.

#### PET scan protocol

PET images were acquired with a Siemens ECAT EXACT HR + scanner (CTI PET systems, Inc., Knoxville, TN, USA) with an axial field of view of 155 mm, providing 63 contiguous 2.46-mm slices with 5.6-mm transaxial and 5.4-mm axial resolution. After a 10-min transmission scan for tissue attenuation correction, infusion of [^11^C]PIB (about 370 MBq in 5 mL for 1 min) began. A PET scan in 3D mode was started after the arrival of tracer to the brain (approximately 30 s after the beginning of tracer infusion). The dynamic scans consisted of 19 frames (3 × 20 s, 3 × 40 s, 1 × 1 min, 2 × 3 min, 5 × 6 min, and 5 × 10 min) with the total scan duration of 90 min. All data processing and image reconstruction were performed using standard Siemens software, which included scatter correction, randoms, and dead time correction.

#### Region-of-interest delineation

Region-of-interest (ROI) analysis was performed using the PMOD software package (PMOD version 3.2; Technologies Ltd., Adliswil, Switzerland). The [^11^C]PIB PET images were co-registered to *T*_1_ weighted images in each subject. The following 15 ROIs were drawn manually on *T*_1_ weighted images: frontal, mesial temporal, lateral temporal, parietal, occipital, anterior cingulate, and posterior cingulate cortices in both hemispheres as well as the reference tissue (gray matter of cerebellum). ROIs were transferred to co-registered [^11^C]PIB PET images, and time-activity curves (TACs) were obtained in those brain regions.

#### Input function measurement

During PET scan, arterial blood was collected from radial artery, starting 6 s (transit delay at the blood sampling site) after the beginning of PET scan to 85 min post injection (10 × 10 s, 1 × 30 s, 9 × 2 min, 6 × 10 min, and 1 × 5 min; 27 samples). Radioactive metabolites were analyzed by a radio-thin layer chromatography (TLC) method [12], with a TLC-developing solvent (ethyl acetate/*n*-hexane = 2:1 vols). The metabolite-corrected radioactivity as well as total radioactivity in blood plasma was fitted to a mono-exponential saturation function during infusion (0 to 1 min) and the sum of three-exponential functions after the end of infusion (1 to 85 min) [12].

#### 4P and 3P + analyses (arterial-plasma input)

Brain regional TACs were analyzed by the weighted NLS method under positive constraint of all *k*_*i*_ with metabolite-corrected input function to afford *K*_1_ to *k*_4_ estimates in 4P analysis (scan time of 90 min) and *K*_1_ to *k*_3_ estimates in 3P + analysis (40 min). Correction was made for blood-pool (5%) radioactivity in brain tissue [14]. Custom software operating in IDL software (version 6.0; Jicoux Datasystems, Inc., Tokyo, Japan) environment was used for the compartment model analysis.

#### 3P++ analysis (reference tissue input)

For successful convergence in NLS optimization using Equation 1, we fixed *k*_2r_ to 0.178/min (mean cerebellar *k*_2_ value by 40-min 3P + analysis; *N* = 30; SD = 0.034). Based on Equation 1 and cerebellar TAC with a fixed *k*_2r_ value, the time-integral of *C*_*r*_(*t*) (the second term on the right side of Equation 1) and the convolution integral (the third term) were calculated numerically without data interpolation for each scan mid-times during 0 to 40 min, and the three parameters *R*_1_, *k*_2_, and *k*_3_ were estimated.

### Simulation study

#### Generation of error-added TACs for Monte Carlo simulation

The error-free, baseline TACs (19 frames/90 min) simulating the target ROI of the NC and AD subjects were generated by using the 4P model with parameter set (*K*_1_ = 0.180 mL/g/min, *k*_2_ = 0.180/min, *k*_3_ = 0.018 and 0.036/min for the NC and AD subjects, respectively, and *k*_4_ = 0.018/min; typical values for [^11^C]PIB) and averaged (*N* = 20) input function of [^11^C]PIB. The reference ROI was the same between NC and AD subjects and was generated by using the 2P model with parameter set (*K*_1_ = 0.180 mL/g/min, *k*_2_ = 0.180/min) and the same input function as above. The error-added TACs for simulation were generated according to the following formula [18]:

(2) $$\begin{array}{l} \mathrm{Error}‒\mathrm{added}\;C_{i}=C_{i}+\mathrm{Rand}\times \sigma \left(C_{i}\right),\\ \;\;\;\sigma \left(C_{i}\right)\;=\;\epsilon \sqrt{\frac{C_{i}}{\Delta t_{i}\times \exp\left(-\lambda t_{i}\right)},} \end{array}$$

where *C*_*i*_ is noise-free simulated radioactivity concentration at frame number *i*, Rand is a random number from a Gaussian distribution with a mean 0 and variance 1, *ϵ* is a scaling factor that determines the noise level, Δ*t*_*i*_ is scan duration of frame number *i*, *t*_*i*_ is mid-scan time of frame number *i*, and *λ* is ^11^C decay constant. In all Monte Carlo simulations, a data set of 100 noise-added TACs was analyzed with weighted NLS, using a relative weight *w*_*i*_:

(3) $$w_{i}=\mathrm{constant}\times \frac{\Delta t_{i}\times \exp\left(-\lambda t_{i}\right)}{C_{i}}.$$

#### Effects of PET noise on 4P, 3P+, and 3P++ analyses

Five levels of PET noise (0.025, 0.05, 0.1, 0.2, and 0.3; *ϵ* in Equation 2, relative values empirically determined) were added to the baseline TACs of the target ROI of the NC subjects. From 100 error-added TACs for each PET noise level, 100 *k*_3_ values were estimated using 90-min 4P, 40-min 3P+, and 3P++ analyses. Coefficient-of-variation (CV) of *k*_3_ was calculated as CV (%) = (SD/mean) × 100. In the following simulations, the PET noise was fixed at 0.1.

#### Effects of K_1_ change in target ROI on 4P, 3P+, and 3P++ analyses

Simulated target TACs were generated by 4P model with five different *K*_1_ values (0.12, 0.15, 0.18, 0.21, and 0.24 mL/g/min) and fixed *k*_3_ (0.018/min) and *k*_4_ (0.018/min). The value of *K*_1_/*k*_2_ was fixed at 1. The range of *K*_1_ was determined with clinically measured *K*_1_ for [^11^C]PIB (0.177 ± 0.31 in NC group and 0.168 ± 0.30 in AD group; 90-min 4P analysis). Reference TAC was the same as baseline reference TAC. The *k*_3_ bias in 90-min 4P, 40-min 3P+, and 3P++ analyses relative to the true *k*_3_ (0.018/min) was calculated as bias (%) = (estimated *k*_3_/true *k*_3_ - 1) × 100.

#### Effects of k_2_ or k_3_ change in reference ROI on 3P++ analysis

In 3P++ analysis, *k*_3r_ was assumed to be 0 and *k*_2r_ was fixed as an empirical constant. The effects of *k*_2r_ or *k*_3r_ change were investigated as follows. The error-added target TACs were generated by 4P model with two different *k*_3_ values (0.018/min for NC and 0.036/min for AD); other parameters were the same as the baseline target TAC. The error-added reference TACs were generated by 2P model with five different *k*_2_ (0.12, 0.15, 0.18, 0.21, and 0.24/min) and fixed *K*_1_ values (0.18 mL/g/min). Another set of simulated reference TACs was generated by 3P model (not 2P model) with five different *k*_3_ (0, 0.002, 0.004, 0.006, and 0.008/min) and fixed *K*_1_ (0.18 mL/g/min) and *k*_2_ (0.18/min). The *k*_3_ bias in 3P++ analysis was expressed relative to 3P + analysis as bias (%) = (3P++ *k*_3_/3P + *k*_3_ - 1) × 100.

Although *k*_3r_ was assumed to be 0 in Equation 1, each subject may have different *k*_3r_ values that deviated from 0. In simulations to investigate the effect of the individual *k*_3r_ variation on 3P++ analysis, we defined the *k*_3_ value empirically corrected for nonzero *k*_3r_ as follows: *k*_3_′ = *k*_3_ + *k*_3r_, where *k*_3_ is the *k*_3_ estimate of target ROI by 3P++ analysis and *k*_3r_ is the *k*_3_ estimate of reference ROI by 3P + analysis (true reference *k*_3_). Bias in 3P++ *k*_3_′ relative to 3P + *k*_3_ was compared with the bias in 3P++ *k*_3_ to 3P + *k*_3_.

## Results

### Goodness of model fits in 3P++ analysis

**Figure 1:**
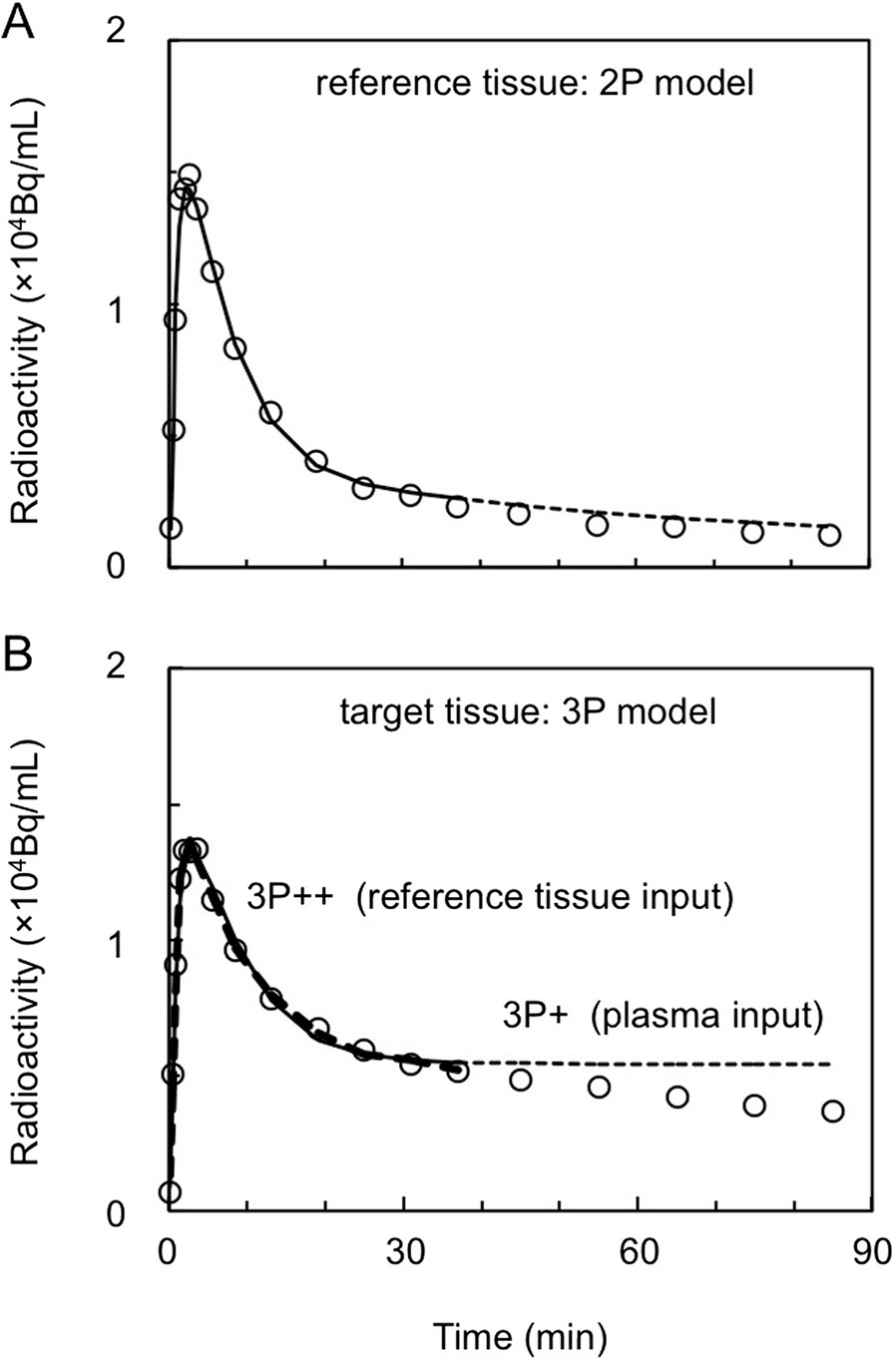
**Reference and target tissue TACs in [**^**11**^**C]PIB PET. (A)** Cerebellar (reference tissue) data (open circle) up to 90 min in one AD subject and the fit of 40-min data to the 2P model (solid line). **(B)** Cerebral cortical (target tissue) data (open circle) in the same subject and the fits of 40-min data to the 3P model with arterial-plasma input (3P + analysis; solid line) or reference tissue input (3P++ analysis; dashed line). The dotted lines in **(A)** and **(B)** indicate the extension of the solid line from 40 to 90 min.

Figure 1A shows an example of the curve fitting of [^11^C]PIB cerebellar TAC data to the 2P model, where a good fit is seen during 0 to 40 min after tracer injection. Figure 1B shows the fits of cerebral cortical TAC data (0 to 40 min) to the 3P + and 3P++ models. The goodness-of-fit by 3P++ model (reference tissue input) is almost indistinguishable from that by 3P + model (arterial-plasma input). Kinetic parameters (*K*_1_ = 0.161 mL/g/min, *k*_2_ = 0.167/min and *k*_3_ = 0.015/min) were estimated in 3P + analysis and *R*_1_ = 0.897, *k*_2_ = 0.158/min and *k*_3_ = 0.011/min in 3P++ analysis.

### Intra-subject k_3_ correlation

**Figure 2:**
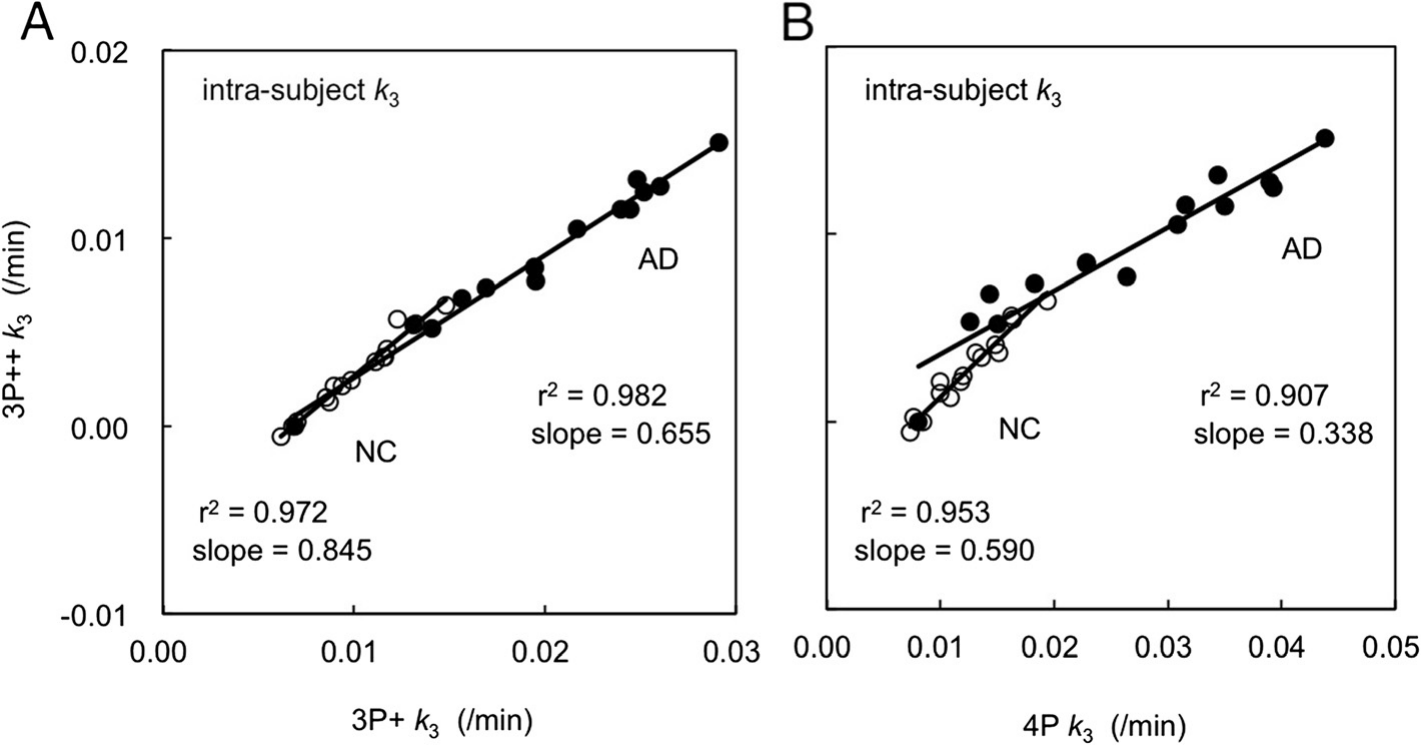
**Intra-subject correlation of *k***_**3 **_**for 15 ROIs in [**^**11**^**C]PIB PET.** The results in 40-min 3P++ (*Y*-axis) vs. 3P + (*X*-axis) analyses **(A)** and 40-min 3P++ vs. 90-min 4P analyses **(B)** with one NC subject (open circle) and one AD subject (closed circle) are shown.

Figure 2A is an example of the intra-subject *k*_3_ correlation between 40-min 3P + (*X*-axis) and 3P++ (*Y*-axis) analyses, where the *k*_3_ values of 15 ROIs, including the cerebellum (reference tissue in 3P++ analysis) from one particular NC subject or one particular AD subject, are shown. The regression lines and the coefficients of determination are *Y* = 0.845*X* - 0.006 (*r*^2^ = 0.972) for the NC subject and *Y* = 0.655*X* - 0.004 (*r*^2^ = 0.982) for the AD subject. Cerebellar *k*_3_ values for both subjects are naturally calculated to be 0 in the 3P++ analysis. The slopes of the regression lines indicate the presence of negative bias in the 3P++ against the 3P + analysis.

Figure 2B shows the *k*_3_ correlation between 90-min 4P (*X*-axis) and 40-min 3P++ (*Y*-axis) analyses in the same subjects. The regression lines are *Y* = 0.590*X* - 0.005 (*r*^2^ = 0.953) for the NC subject and *Y* = 0.338*X* + 0.000 (*r*^2^ = 0.907) for the AD subject. When the cerebellar data (*X* = 0.008, *Y* = 0.000) was removed from calculation for the AD subject, the regression line became *Y* = 0.295*X* - 0.002 with slightly larger *r*^2^ (0.935; not shown in the figure). The slopes of the regression lines show that *k*_3_ bias in 3P++ against 4P analysis is larger than that against 3P + analysis.

### Inter-subject k_3_ correlation

**Figure 3:**
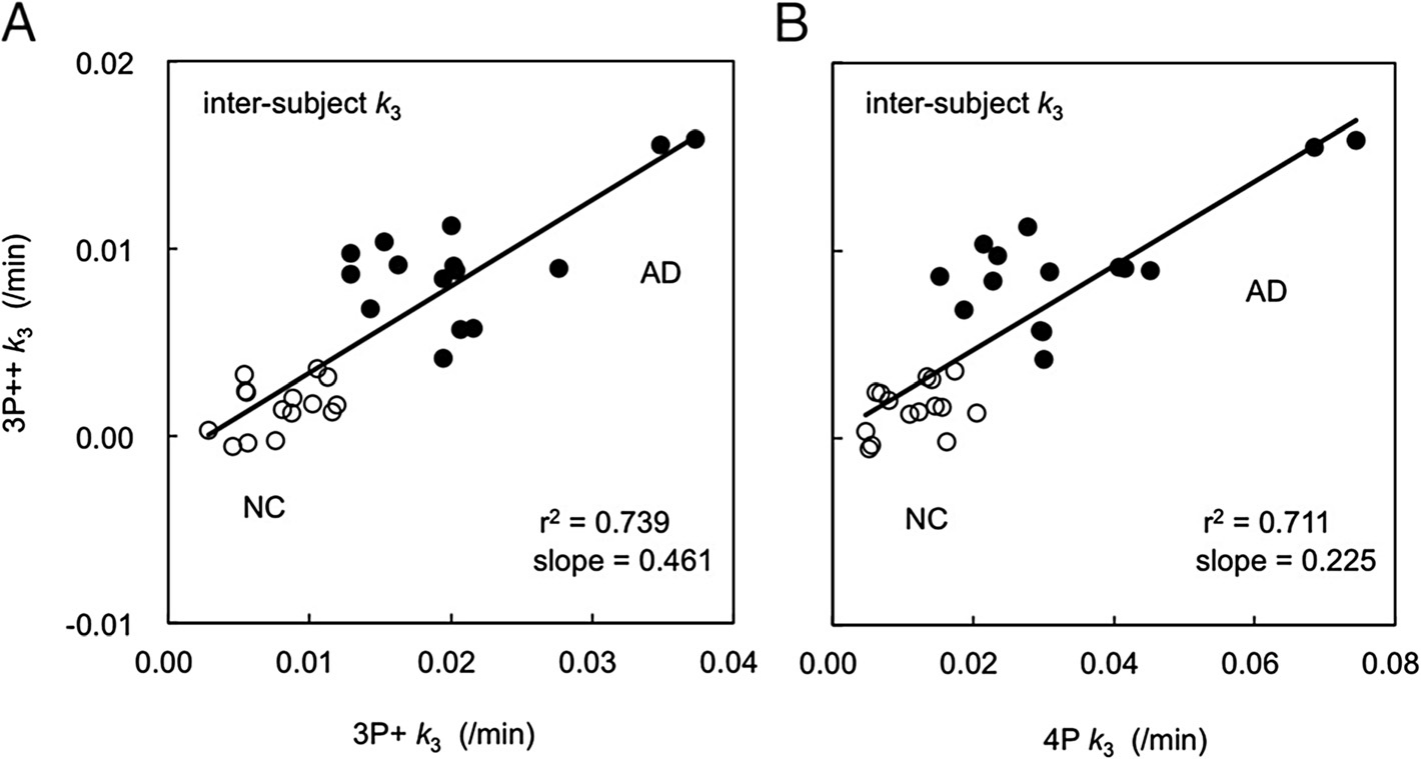
**Inter-subject correlations of left lateral temporal *k***_**3 **_**in [**^**11**^**C]PIB PET.** The results in 40-min 3P++ (*Y*-axis) vs. 3P + (*X*-axis) analyses **(A)** and 40-min 3P++ vs. 90-min 4P analyses **(B)** with 30 subjects (15 NC, open circle; 15 AD, closed circle) are shown.

Figure 3A shows an example of the inter-subject *k*_3_ correlation, where *k*_3_ values for the left lateral temporal cortex from 30 subjects (15 NC + 15 AD) are compared between 40-min 3P + (*X*-axis) and 3P++ (*Y*-axis) analyses. The regression lines are *Y* = 0.461*X* - 0.001 (*r*^2^ = 0.739) for all 30 subjects, *Y* = 0.178*X* + 0.000 (*r*^2^ = 0.151) for the NC group alone, and *Y* = 0.286*X* + 0.003 (*r*^2^ = 0.411) for the AD group alone; the latter two lines are not shown in the figure. The slopes of the regression lines also indicate the presence of negative biases in 3P++ against 3P + analysis.

Figure 3B shows the inter-subject correlation of left lateral temporal *k*_3_ between 90-min 4P (*X*-axis) and 40-min 3P++ (*Y*-axis) analyses, where the regression line is *Y* = 0.225*X* + 0.000 (*r*^2^ = 0.711) for all subjects. The lines of *Y* = 0.090*X* + 0.001 (*r*^2^ = 0.122) for the NC group alone and *Y* = 0.135*X* + 0.005 (*r*^2^ = 0.513) for the AD group alone were also calculated. The slopes of the regression lines show larger negative *k*_3_ biases in 3P++ against 4P analysis than that shown in Figure 3A. The results in other cerebral regions were essentially the same as those in the left lateral temporal cortex.

### Simulation on the effects of PET noise on k_3_ CV

**Figure 4:**
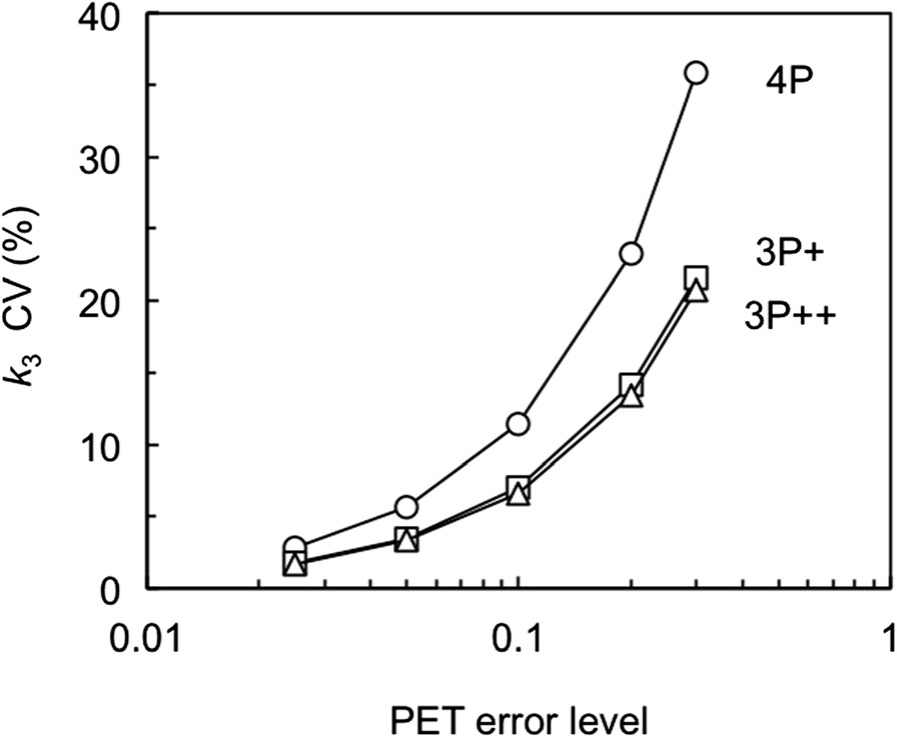
**Effects of PET noise on CV of *k***_**3**_**.** The results in 40-min 3P++ (open triangle), 40-min 3P + (open square), and 90-min 4P (open circle) analyses are shown. Five different PET noises (0.025 to 0.3) were added to the [^11^C]PIB baseline TACs of the target ROI of the NC subjects. CV of *k*_3_ was calculated from 100 *k*_3_ estimates as CV (%) = (SD/mean) × 100.

Figure 4 compares the noise sensitivity of *k*_3_ estimates among the 90-min 4P, 40-min 3P+, and 3P++ analyses. In all three analyses, the *k*_3_ CVs increased as the PET error became larger. The *k*_3_ CV in 3P++ analysis was comparable to that in 3P + analysis and lower than that in 4P analysis; for example, *k*_3_ CVs at 0.1 of noise level were 6.6% in 3P++, 7.0% in 3P+, and 11.4% in 4P analyses.

### Simulation on the effects of target K_1_ change on k_3_ bias

**Figure 5:**
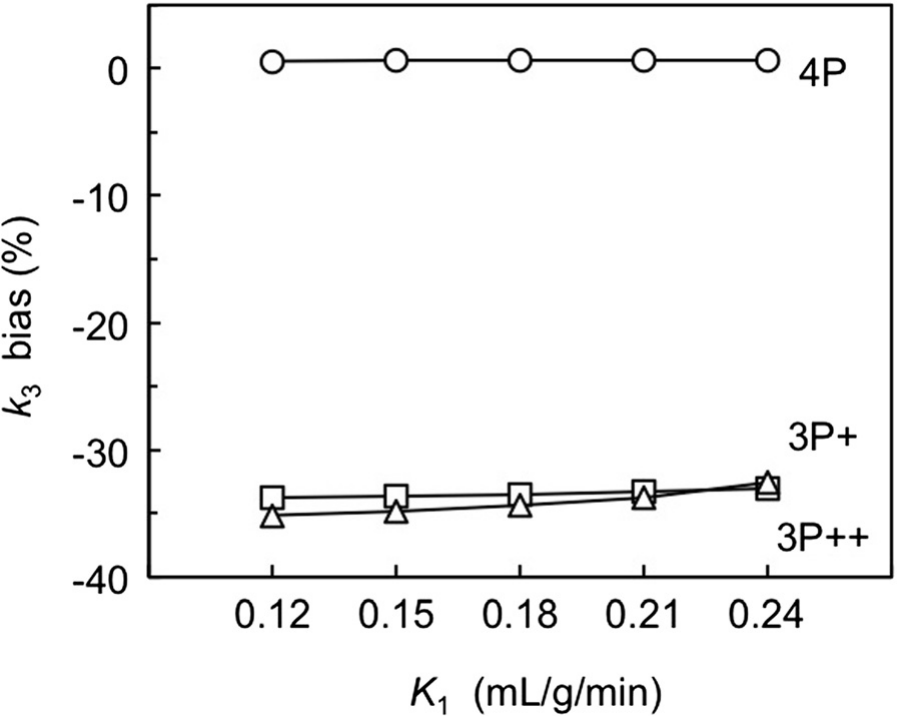
**Effects of *K***_**1 **_**change in the target region on *k***_**3 **_**bias.** The results in 40-min 3P++ (open triangle), 40-min 3P + (open square) and 90-min 4P (open circle) analyses are shown. Simulated target TACs were generated by 4P model with five different *K*_1_ values (0.12 to 0.24 mL/g/min). The *k*_3_ bias was calculated as bias (%) = (estimated *k*_3_/true *k*_3_ – 1) × 100.

Figure 5 shows the effects of *K*_1_ change in the target ROI on the *k*_3_ biases in the 90-min 4P, 40-min 3P+, and 3P++ analyses. The 4P analysis remained almost bias-free (+0.6%) within *K*_1_ from 0.12 to 0.24 mL/g/min. 3P + and 3P++ analyses showed larger negative biases (-33% to -34% bias in 3P + and -33% to -35% bias in 3P++) compared with 4P analysis. Although 3P++ analysis showed slightly larger *k*_3_ bias than 3P + analysis when *K*_1_ was low (0.12 mL/g/min), *k*_3_ bias in 3P++ analysis was almost the same as 3P + analysis.

### Simulation on the effects of k_2r_ change on 3P++ analysis

**Figure 6:**
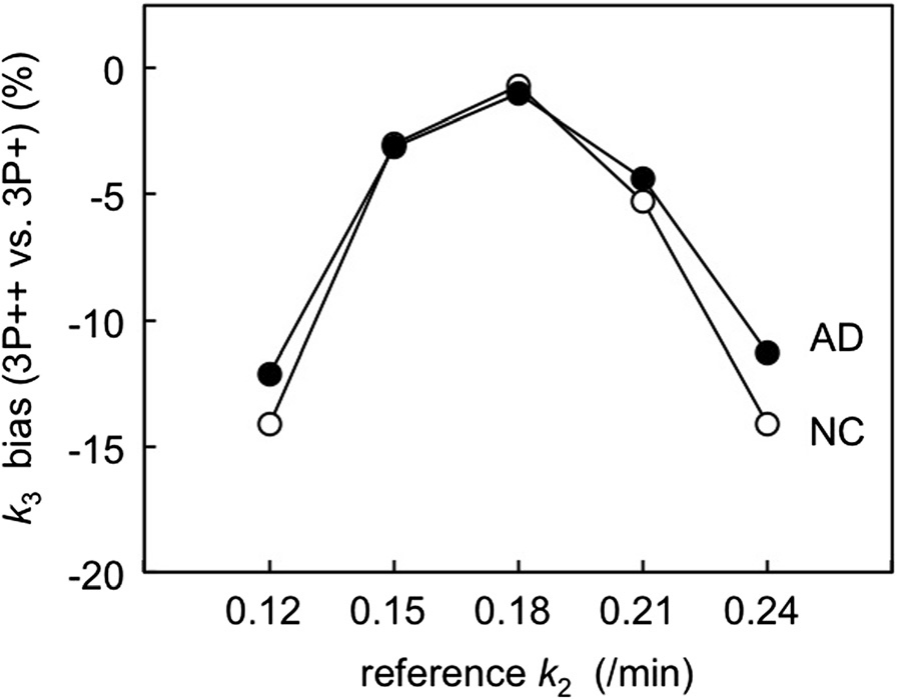
**Effects of *k***_**2 **_**change in the reference region on *k***_**3 **_**bias in 40-min 3P++ analysis.** Simulated target TACs were generated by 4P model with two different *k*_3_ values (0.018/min for NC, open circle; 0.036/min for AD, closed circle). Simulated reference TACs were generated by 2P model with five different *k*_2_ values (0.12 to 0.24/min). The *k*_3_ bias in 3P++ analysis was expressed relative to 3P + analysis as bias (%) = (3P++ *k*_3_/3P + *k*_3_ – 1) × 100.

In 3P++ analysis (Equation 1), *k*_2r_ was fixed at 0.178/min, though *k*_2r_ was not always the same among subjects (CV = 19%). Figure 6 shows the effects of individual *k*_2r_ change in 40-min 3P++ analysis. When *k*_2r_ was equal to the fixed value (0.18/min), 3P++ analysis was bias-free, relative to 3P + analysis. However, when *k*_2r_ was different from the fixed value, 3P++ analysis showed a negative *k*_3_ bias relative to 3P + *k*_3_. The *k*_2r_ effects were similar between NC ROI (*k*_3_ = 0.018/min) and AD ROI (*k*_3_ = 0.036/min); for example, the biases were -14.1% for NC and -12.1% for AD at *k*_2r_ = 0.12/min and -14.1% for NC and -11.3% for AD at *k*_2r_ = 0.24/min.

### Simulation on the effects of k_3r_ change on 3P++ analysis

In 3P++ analysis we assume that *k*_3r_ = 0, that is, specific binding is negligible in the reference tissue. However, in all subjects examined, this assumption did not hold: the *k*_3r_ values in 40-min 3P + analysis were 0.008 ± 0.004/min in the AD group, 0.007 ± 0.002/min in the NC group, and 0.007 ± 0.003/min in the AD + NC group.

**Figure 7:**
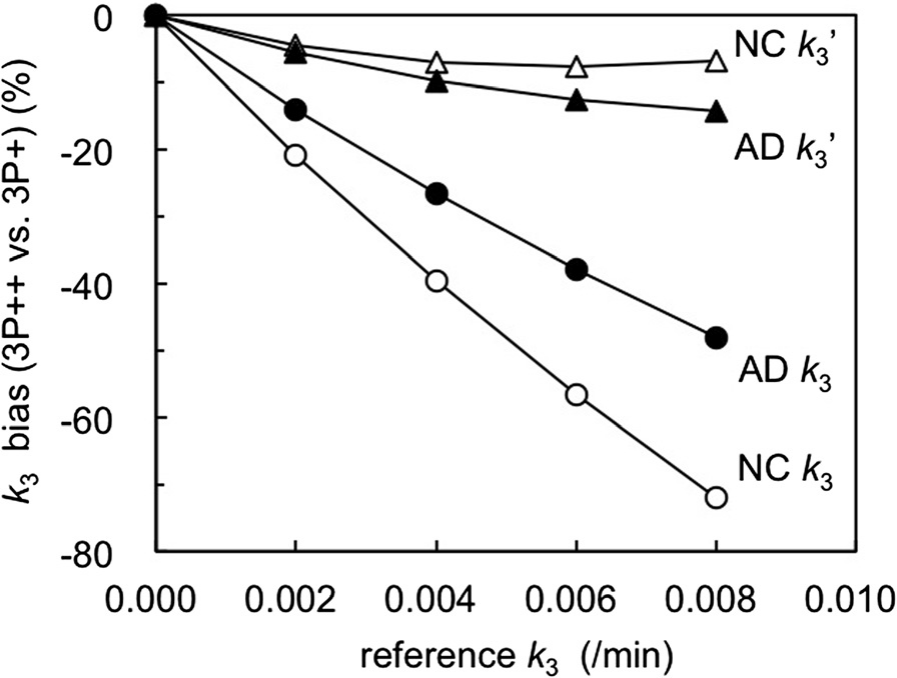
**Effects of *k***_**3 **_**change in the reference region on *k***_**3 **_**bias in 40-min 3P++ analysis.** Simulated target TACs were generated by 4P model with two different *k*_3_ values (0.018/min for NC, open circle; 0.036/min for AD, closed circle). Simulated reference TACs were generated by 3P model with five different *k*_3_ values (0 to 0.008/min). The *k*_3_ bias in 3P++ analysis was expressed relative to 3P + analysis as bias (%) = (3P++ *k*_3_/3P + *k*_3_ – 1) × 100. Effects on bias in 3P++ *k*_3_′ relative to 3P + *k*_3_ are also shown (NC, open triangle; AD, closed triangle), where 3P++ *k*_3_′ was calculated as (3P++ *k*_3_′) = (3P++ *k*_3_) + reference *k*_3_.

Figure 7 shows the effects of individual *k*_3r_ change (0 to 0.008/min) on 40-min 3P++ analysis. When *k*_3r_ was 0, 3P++ analysis was bias-free, relative to 3P + analysis. The *k*_3_ biases (negative biases) increased as *k*_3r_ increased: -38% for NC and -27% for AD at *k*_3r_ = 0.004/min and -70% for NC and -48% for AD at *k*_3r_ = 0.008/min. The NC ROI (*k*_3_ = 0.018/min) showed larger biases than the AD ROI (*k*_3_ = 0.036/min). Figure 7 also shows the results of the simulation study on the relationship between 3P++ *k*_3_′ and 3P + *k*_3_, where 3P++ *k*_3_ was empirically corrected with individual *k*_3r_. In this case, negative bias in 3P++ *k*_3_′ was significantly decreased compared to that in 3P++ *k*_3_; for example, bias was decreased from -70% to -7% for NC, and from -48% to -15% for AD at *k*_3r_ = 0.008/min.

**Figure 8:**
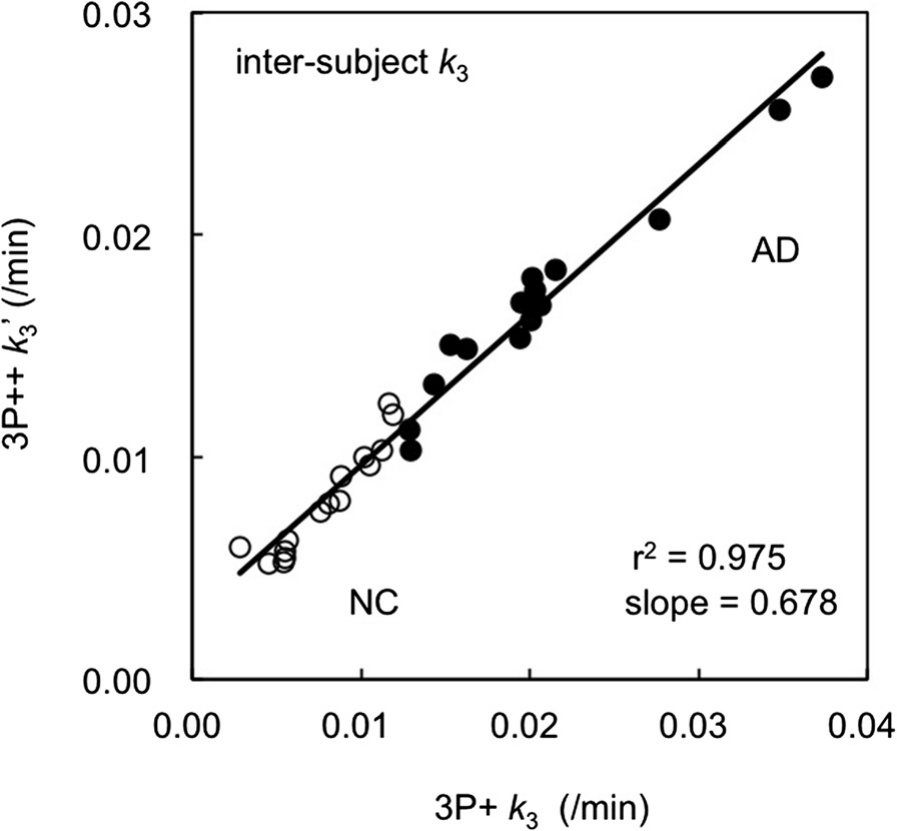
**Inter-subject correlation of left lateral temporal *k***_**3 **_**in [**^**11**^**C]PIB PET.** The result in 40-min 3P++ (*Y*-axis) vs. 3P + (*X*-axis) analyses with 30 subjects (15 NC, open circle; 15 AD, closed circle) is shown. The *k*_3_ estimates were empirically corrected as (3P++ *k*_3_′) = (3P++ *k*_3_) + (individual cerebellar *k*_3_ by 3P+).

Figure 8 shows the correlation between 3P++ *k*_3_′ and 3P + *k*_3_ using the same data as in Figure 3A, where 3P++ *k*_3_ in Figure 3A was replaced by 3P++ *k*_3_′. The regression line was *Y* = 0.678*X* + 0.003 (*r*^2^ = 0.975) for all subjects, where *X* = 3P + *k*_3_ and *Y* = 3P++ *k*_3_′. The lines of *Y* = 0.798*X* + 0.002 (*r*^2^ = 0.897) for the NC group alone and *Y* = 0.620*X* + 0.004 (*r*^2^ = 0.960) for the AD group alone were also calculated. The determination coefficient was increased by this correction from 0.739 to 0.975. The slope of the regression line was also increased from 0.461 (Figure 3A) to 0.678 (Figure 8), which showed the reduction of negative bias in 3P++ analysis.

## Discussion

### Theoretical basis and merits of 3P++ analysis

The previous 3P + analysis allowed for estimating *k*_3_ of moderately reversible ligands, where the 3P model was applied to early-phase (up to 30 to 40 min) PET data with arterial input function [13]. It was reported that when the 3P model was applied to 60-min PET scan data from [^11^C]PIB (*k*_4_ = 0.018/min) as a moderately reversible ligand, only a poor model fit was obtained [19]. Previous simulation studies on [^11^C]PIB using information density theory suggested that scan time reduction to 40 min would be necessary to obtain a good fit to the 3P model [13].

When 3P + or 3P++ analysis can be applied to a ligand, such ligand is specified as a moderately reversible ligand. This applicability is determined by the information function curves of *k*_3_ and *k*_4_[13], and thus is dependent on the scan time as well as *k*_3_ and *k*_4_ values of the ligand in a ROI. Differentiation of a moderately reversible ligand from general reversible ligands is somewhat arbitrary, though we conveniently defined this with the *k*_4_ value (≤0.03/min) in this study.

In the present study, the 3P + plasma input model was extended to the 3P++ reference tissue input model. The 3P++ analysis has three merits over previous methods. First, the PET scan time is short, usually less than 40 min, which may be important in PET studies with elderly or demented subjects. Secondly, the target parameter *k*_3_ can be isolated from the other model parameters. Thirdly, neither arterial cannulation nor labor-intensive measurements of labeled metabolites are required.

One of the conventional models for the estimation of binding of [^11^C]PIB is the Logan plot analysis [2], which employs data of long duration (more than 60 min). Noninvasive Logan analysis (distribution volume ratio) [6] requires late-phase (equilibrium-phase) PET data, whereas late-phase data are not necessary for 3P++ analysis. In the noninvasive Logan model or simplified reference tissue model [8], the *K*_1_-to-*k*_2_ ratio in the target and reference tissues is assumed to be equal. 3P++ analysis does not require such an assumption. Since 3P++ analysis is a kind of irreversible-model analysis, *K*_1_ (*R*_1_) and *k*_3_ can be independently estimated (*k*_2_ must be fixed to a certain constant).

### Noise sensitivity of 3P++ analysis

Loss of PET data in short-scan 3P++ and 3P + analyses might be considered to deteriorate the precision of the *k*_3_ estimate. In the present simulation for noise sensitivity, *k*_3_ CV values in 40-min 3P++ and 3P + analyses were lower than (almost three fifths of) that in 90-min 4P analysis (Figure 4), which was in accordance with the previous report [13]. It is considered that the loss of PET data may be compensated for by the reduction in the number of free parameters from four in the 4P model to three in the 3P + and 3P++ models.

### K_1_ effect on 3P++ analysis

In the *K*_1_ simulation, the stableness of *k*_3_ estimation in changes of cerebral blood flow was investigated. The magnitudes of *k*_3_ bias were independent of the *K*_1_ change, ranging from 0.12 to 0.24 mL/g/min, in 3P++, 3P+, and 4P analyses (Figure 5). The 3P++ as well as 3P + and 4P analyses were less affected by *K*_1_, which is owing to the capability of isolating the *k*_3_ estimation. The 40-min 3P + analysis showed -33% *k*_3_ bias relative to 90-min 4P analysis, which is in accordance with the previous report [13]. In this *K*_1_ simulation, 3P++ *k*_3_ showed negligible bias relative to 3P + *k*_3_. These results suggested that in 3P++ analysis, the effects of ignoring vascular volume as well as numerical integration error due to discrete time points were not significant.

### Causes of negative k_3_ bias in 3P++ analysis

Firstly, the *k*_3_ bias in 3P++ analysis originates from 3P model approximation. Our previous simulation study [13] showed that the 3P + analysis with 28-min scan had large negative *k*_3_ bias relative to 4P analysis with 90-min scan; for example, there was about -22% to -24% bias to true *k*_3_ (4P *k*_3_) ranging from 0.01 to 0.04/min including NC and AD *k*_3_. 3P++ analysis showed further negative *k*_3_ bias relative to 3P + analysis due to the following two reasons.

Secondly, the bias is due to individual *k*_2r_ change from the fixed value in Equation 1. In 3P++ analysis, we also assumed that *k*_2_ in the reference tissue was constant and was fixed at 0.178/min, which was the average *k*_2_ value with the 3P + model. In simulation, negative *k*_3_ bias was predicted when *k*_2r_ was larger or smaller than fixed *k*_2_ (Figure 6). Each subject in the NC and AD groups had different *k*_2_ values in the reference tissue, and it is considered that such biological variance as for reference tissue may result in a negative *k*_3_ bias in 3P++ analysis, relative to 3P + analysis for [^11^C]PIB.

Thirdly, the bias is due to the discrepancy between the model assumption and the actual reference ROI. The basic assumption (assumption 3) in 3P++ analysis is *k*_3r_ = 0. The working equation of 3P++ analysis (Equation 1) is derived under this assumption, and reference *k*_3_ is naturally calculated to be 0. However, in 3P + analysis with [^11^C]PIB, the cerebellum showed nonzero *k*_3_ (0.007 ± 0.003/min in all 30 subjects). Thus, 3P++ *k*_3_ is expected to be underestimated. Simulation studies showed that 3P++ analysis was bias-free for ideal reference with zero *k*_3_ and that *k*_3_ bias became larger as *k*_3r_ increased (Figure 7). When *k*_3_ was replaced by *k*_3_′, negative bias was significantly decreased in the simulation (Figure 7), as well as the slope of the regression line between 3P++ and 3P + analyses being increased from 0.461 (Figure 3A) to 0.678 (Figure 8), which also suggested that nonzero *k*_3r_ caused underestimation of 3P++ *k*_3_.

### Correlation of k_3_ between 3P++ and 3P + analyses

Strong intra-subject *k*_3_ correlation was shown between 3P++ and 3P + analyses, and the rank-order of *k*_3_ was almost the same between the two analyses (Figure 2A), suggesting the stability of both 3P++ and 3P + analyses.

The inter-subject *k*_3_ correlation (*r*^2^; Figure 3A) was significantly lower than the intra-subject correlation (Figure 2A). Such a lower inter-subject *k*_3_ correlation can be partly explained by the sample variance of cerebellar *k*_3_. In order to explain this, *k*_3_′ was calculated for each subject. When *k*_3_ was replaced by *k*_3_′, the determination coefficient between 3P++ and 3P + analyses was increased from 0.739 (Figure 3A) to 0.975 (Figure 8); the latter is comparable to *r*^2^ of the intra-subject *k*_3_ correlation (0.982; Figure 2A).

Such an estimation of parameter *k*_3_′ is not always practical, as 3P + analysis with arterial input function is necessary for individual cerebellar *k*_3_ estimation. However, these results suggest that the lower *r*^2^ in the inter-subject correlation compared with the intra-subject correlation is due to the sample variance of cerebellar *k*_3_ and that 3P++ analysis itself is robust, as far as the reference is ideal.

Practically, the use of mean *k*_3r_ may be meaningful. When target *k*_3_ is empirically corrected as corrected *k*_3_ = estimated *k*_3_ + mean cerebellar *k*_3_, the absolute bias in target *k*_3_ would decrease. However, the precision of target *k*_3_ would not necessarily be improved owing to the variance of individual *k*_3r_.

In addition to the nonzero effect of *k*_3r_, inter-subject variation of *k*_2r_ from the fixed value (*k*_2_ = 0.178/min) may also produce individually different *k*_3_ bias in 3P++ analysis, resulting in lower inter-subject *k*_3_ correlation between 3P + and 3P++ analyses.

### Limitations of 3P++ analysis

When 3P++ analysis was applied to [^11^C]PIB as an example of moderately reversible ligands, a somewhat lower inter-subject *k*_3_ correlation (*r*^2^ = 0.739 or 0.711; Figure 3A or Figure 3B) was shown between the 3P++ and 3P + or 4P analyses, respectively, across a *k*_3_ range including NC and AD (3P + *k*_3_, 0.004 to 0.040/min). The rank order of 3P++ *k*_3_ also differed considerably from 3P + *k*_3_ or 4P *k*_3_. These results were mainly due to nonzero *k*_3r_ and the sample variance of both *k*_2r_ and *k*_3r_ as described above. The negative *k*_3_ bias (3P++ vs. 3P+) was larger in NC ROI (-70%) than in AD ROI (-48%) when *k*_3r_ = 0.008/min (Figure 7). The previous report showed that the difference in *k*_3_ bias (28-min 3P + vs. 90-min 4P) was small between NC ROI (-23%) and AD ROI (-24%) [13]. Therefore, the *k*_3_ value in 3P++ analysis may be somewhat underestimated in the ROI with lower amyloid deposition compared to 3P + or 4P analysis.

In [^11^C]PIB PET, 3P++ analysis may be inadequate for inter-subject *k*_3_ comparison and useful only for intra-subject (inter-ROI) comparison or pre- vs. post-comparison in the same subject. 3P++ analysis would be more suitable for such reversible ligands that have moderate *k*_4_ and reference tissue without specific binding.

## Conclusions

The 3P++ analysis is a *k*_3_ estimation method for moderately reversible PET ligands with a short scan time such as 40 min and without arterial blood sampling. Although the applicability of 3P++ method to [^11^C]PIB PET may be restricted to intra-subject comparison, 3P++ analysis itself is robust. The 3P++ method would be useful for PET study with non-highly reversible ligands, as far as the reference tissue without specific binding is available.

## Competing interests

The authors declare that they have no competing interests.

## Authors' contributions

KS participated in clinical PET study and the simulation study, and drafted the manuscript. KF conceived of the study, participated in the simulation study, and helped to draft the manuscript. HS (Shinotoh), HS (Shimada), SH, and NT participated in clinical PET study and contributed to the discussion. TS, TI, and HI supervised the design and coordination of the study. All authors read and approved the final manuscript.
